# Editorial: Beyond contraception - advancing research and innovations in sexual and reproductive health to better meet the needs of women in Low-and middle-income countries

**DOI:** 10.3389/frph.2026.1814109

**Published:** 2026-03-13

**Authors:** Amanda Cordova-Gomez, Sesilia Shirima, Emeka Nwachukwu

**Affiliations:** 1 Local2Global Lab, Silver Spring, MD, United States; 2 Young & Alive Initiative, Dar es Salaam, Tanzania; 3 Innovate Health Research Initiative, Abuja, Nigeria

**Keywords:** endometriosis, fibroids, infertility, PCOS, SRH, innovation, women's health

**Editorial on the Research Topic**
Beyond contraception - advancing research and innovations in sexual and reproductive health to better meet the needs of women in low-and middle-income countries

Historically, women's health has received limited and uneven investment in research, resulting in substantial gaps in the evidence base across conditions that disproportionately affect women across the life course. Only a small fraction of global health research and development funding is directed toward women's health, much of it concentrated in narrow domains, while highly prevalent gynecological conditions (including endometriosis, chronic pelvic pain, uterine fibroids and polycystic ovary syndrome) remain comparatively underfunded (1–3). These imbalances have constrained scientific understanding, delayed innovation globally, and limited the development of effective, accessible interventions for women worldwide, while innovation ecosystems have remained predominantly anchored in high-income countries and only later extended to low- and middle-income countries (LMICs) contexts through downstream implementation.

Despite growing global attention to women's health, inequities persist in whose knowledge is funded, produced, published, and valued. Much of the evidence informing health policies, clinical guidelines, and innovation pathways continues to be generated, indexed and interpreted by institutions in high-income countries (HICs), even when the greatest burdens of disease and unmet need are borne by women and girls in LMICs. These disparities reflect not only geographic differences in research output but longstanding asymmetries in funding allocation, authorship, editorial influence, and agenda-setting power (4, 5).

Women in LMICs experience a disproportionate share of preventable morbidity and mortality across sexual and reproductive health, maternal and newborn health, gynecologic conditions, non-communicable diseases, mental health, and exposure to gender-based violence (6). These outcomes are shaped by intersecting social, economic, political, and gendered determinants that vary across settings. Yet research originating in LMICs, particularly work led by local investigators, remains underrepresented in the global literature. Limited access to funding, inequitable research partnerships, publication costs, language bias, and entrenched editorial norms continue to restrict the reach and influence of LMIC-led scholarship.

This special issue responds to these persistent gaps, with a focus on sexual and reproductive health beyond contraception and family planning. While contraceptive access has received sustained global investment, other dimensions of Sexual and Reproductive Health (SRH) that shape women's autonomy, wellbeing, and quality of life remain comparatively underexplored, particularly through locally grounded research perspectives.

This Research Topic's central objective is to strengthen the representation of women's health research conceptualized, led, and authored by researchers in underrepresented settings, alongside increasing the visibility of high-quality data generated within these contexts. Gaps in nationally collected and internationally reported women's health and SRH data across LMICs have limited the completeness of the global evidence base, and subsequently constrained effective policy and programmatic responses (7). By prioritizing locally produced evidence and leadership, this special issue enhances the relevance, contextual validity, and applicability of global women's health knowledge while addressing inequities in data generation and dissemination.

Several contributions are presented in this collection regarding epidemiological and experiential evidence from underrepresented contexts, including African countries. Elwafi et al. report novel data on polycystic ovary syndrome (PCOS) prevalence in Mauritania, identifying a rate of 7.8% alongside population-specific variants in the LHCGR and ESR1 genes that may influence disease susceptibility. This work aligns with global trends and reinforces the importance of genetic research in diverse populations to inform more tailored prevention and treatment strategies.

Eléonore et al. examine primary infertility in Côte d'Ivoire, where infertility represents a substantial proportion of gynecological consultations in clinics. Tubal obstruction emerges as the leading etiology, accounting for 36.5% of cases, alongside a significant psychological burden, with 81.9% of affected women reporting clinically relevant anxiety. In Morocco, Benbella et al. explore the lived experiences of infertile couples, revealing how diagnostic delays, fragmented care pathways, social stigma, and financial hardship intensify distress and limit access to timely treatment. Together, these studies illustrate the intertwined clinical and social impacts of infertility.

Broader evidence is provided by the systematic review by Mesfin et al., which synthesizes 43 studies from LMICs to assess barriers to accessing assisted reproductive technologies (ART). Not surprisingly, high treatment costs emerge as the dominant constraint, followed by infrastructural limitations and socio-cultural challenges, including religious concerns and stigma surrounding ART-conceived children. In addition, the review points to the need for infertility services embedded within existing health systems and supported by sustainable financing mechanisms.

Interestingly beyond infertility, Xie et al. present a meta-analysis of randomized controlled trials demonstrating that Relugolix, a gonadotropin-releasing hormone antagonist, significantly improves pain and quality of life among women with endometriosis as measured by the EHP-30 instrument. This evidence supports expanded non-surgical management options for a condition that remains widely underdiagnosed and undertreated. Five randomized controlled trials met all predefined inclusion criteria and were included in the final meta-analysis, drawing on data from diverse geographic contexts across Africa, Asia, the Americas, and Europe, thereby strengthening the global relevance of the findings.

At the population level, Chen et al. analyzed three decades of Global Burden of Disease data and found a rising burden of uterine fibroids, reflected in both incidence and age-standardized incidence rates, largely driven by population growth. In contrast, they observed a modest decline in the burden of endometriosis, likely associated with broader epidemiological shifts. Drawing on large, multi-regional datasets, including populations from India, China, and the United States, the analysis captures wide variation in incidence and disease patterns across diverse contexts. The rising trends provide important insight for anticipating future reproductive health needs and guiding resource allocation.

Taken together (Table 1), the contributions in this special issue show that women's reproductive health and well-being are shaped by biological processes as well as the social, economic, political, and geographic contexts in which care is accessed. Substantial variation in disease burden, care pathways, and treatment experiences across settings underscores the need for research and health systems that are responsive to local realities rather than built on one-size-fits-all models. The findings also highlight the importance of expanding genetic, epidemiological, and population-level research in diverse communities to strengthen scientific understanding and support more inclusive innovation. At the same time, persistent financial barriers to care emerge as a central constraint, underscoring the need for policy approaches that prioritize affordability, financial protection, and equitable access.

**Table 1:** Summary of studies included in the Research Topic.

Theme/Summary topic	Authors	Setting/Country	Study design	Objectives	Population description
Polycystic ovarian syndrome (PCOS) Prevalence and Genetic Etiology	Elwafi et al.	Nouakchott, Mauritania	Cross-sectional retrospective study with genetic analysis	To evaluate PCOS prevalence and explore gene polymorphisms in the Mauritanian population	2,100 women attending two gynecologic clinics; 164 PCOS patients identified (7.8% prevalence); genetic analysis on 64 PCOS cases
Experiences of Infertility	Benbella et al.	Rabat, Morocco	Qualitative study with in-depth interviews	To explore experiences of infertility among couples who received diagnosis and treatment at Morocco's first public ART center	Couples with infertility receiving care at the ART center of Maternity and Reproductive Health Hospital les Orangers; interviews conducted January-March 2023
Primary Infertility: Epidemiology and Psychology	Eléonore et al.	Abidjan, Côte d'Ivoire	Cross-sectional descriptive study	To improve care and monitoring of women with primary infertility by describing their epidemio-clinical profile and psychological experience	160 women consulting for primary infertility (inability to conceive >1 year) at Angré University Hospital; January 2021-December 2023; mean age 34.46 years
Endometriosis Treatment Efficacy	Xie et al.	Shanghai, China (meta-analysis of global RCTs)	Systematic review and meta-analysis of RCTs	To evaluate Relugolix's comprehensive impact on endometriosis-associated pain and health-related quality of life using EHP-30 questionnaire	Five RCTs of women with endometriosis receiving Relugolix vs. placebo or other comparators; outcomes assessed via EHP-30 questionnaire
ART Access Barriers in Developing Countries	Mesfin et al.	Multiple developing countries (systematic review)	Systematic review	To explore challenges faced by couples in developing countries accessing medically assisted reproductive technology and identify solutions	43 studies from developing countries examining barriers to ART access; searched databases included Ovid MEDLINE, PubMed, Web of Science, Google Scholar
Global Burden of Uterine Fibroids and Endometriosis	Chen et al.	Global, with focus on China, India, USA	Global burden of disease database analysis with predictive modeling	To assess disease burden of uterine fibroids and endometriosis in reproductive-age women (1990–2021) and predict future trends to 2036	Women of reproductive age globally and in China, India, USA; analyzed GBD 2021 data for incidence, ASIR, DALYs, and ASDR

This collection is further strengthened by its methodological breadth, integrating qualitative and quantitative studies, systematic and narrative reviews, and other context-responsive approaches. Together, these methods capture both population-level patterns and lived experiences, offering a more comprehensive understanding of women's reproductive health across diverse settings.

In conclusion, advancing women's health equity will require sustained investment in LMIC-led research ecosystems, partnerships grounded in shared leadership, comprehensive and responsive policies and stronger data systems that reflect women's lived realities across diverse contexts. By amplifying scholarship from underrepresented settings, this special issue contributes to a more inclusive and representative global evidence base in women's health. Crucially, shifting from research conducted about populations to research shaped and led by those working within affected communities transforms not only who produces knowledge, but also the questions prioritized, the methods employed, and the pathways through which evidence informs practice. When local researchers and practitioners guide the evidence agenda, research is more likely to address context-specific needs and generate solutions that are feasible, effective, and sustainable within existing health systems.
